# Testing the Effect of a Smartphone App on Hospital Admissions and Sedentary Behavior in Cardiac Rehabilitation Participants: ToDo-CR Randomized Controlled Trial

**DOI:** 10.2196/48229

**Published:** 2023-10-03

**Authors:** Kacie Patterson, Rachel Davey, Richard Keegan, Theo Niyonsenga, Itismita Mohanty, Sarah Bowen, Elizabeth Regan, Michelle Lander, Sander van Berlo, Nicole Freene

**Affiliations:** 1 Health Research Institute Faculty of Health University of Canberra Bruce, ACT Australia; 2 Research Institute for Sports and Exercise (UCRISE) Faculty of Health University of Canberra Bruce, ACT Australia; 3 National Capital Private Hospital Garran, ACT Australia; 4 Calvary Public Hospital Bruce Bruce, ACT Australia; 5 The Canberra Hospital Garran, ACT Australia; 6 Onmi BV Torenallee, Eindhoven Netherlands; 7 Physiotherapy Faculty of Health University of Canberra Bruce, ACT Australia

**Keywords:** mobile health, secondary prevention, cardiovascular disease, sedentary behavior, hospital admissions, cost-effectiveness, mobile phone

## Abstract

**Background:**

People with coronary heart disease are at an increased risk of morbidity and mortality even if they attend cardiac rehabilitation. High sedentary behavior levels potentially contribute to this morbidity. Smartphone apps may be feasible to facilitate sedentary behavior reductions and lead to reduced health care use.

**Objective:**

We aimed to test the effect of a sedentary behavior change smartphone app (Vire app and ToDo-CR program) as an adjunct to cardiac rehabilitation on hospital admissions and emergency department (ED) presentations over 12 months.

**Methods:**

A multicenter, randomized controlled trial was conducted with 120 participants recruited from 3 cardiac rehabilitation programs. Participants were randomized 1:1 to cardiac rehabilitation plus the fully automated 6-month Vire app and ToDo-CR program (intervention) or usual care (control). The primary outcome was nonelective hospital admissions and ED presentations over 12 months. Secondary outcomes including accelerometer-measured sedentary behavior, BMI, waist circumference, and quality of life were recorded at baseline and 6 and 12 months. Logistic regression models were used to analyze the primary outcome, and linear mixed-effects models were used to analyze secondary outcomes. Data on intervention and hospital admission costs were collected, and the incremental cost-effectiveness ratios (ICERs) were calculated.

**Results:**

Participants were, on average, aged 62 (SD 10) years, and the majority were male (93/120, 77.5%). The intervention group were more likely to experience all-cause (odds ratio [OR] 1.54, 95% CI 0.58-4.10; *P*=.39) and cardiac-related (OR 3.26, 95% CI 0.84-12.55; *P*=.09) hospital admissions and ED presentations (OR 2.07, 95% CI 0.89-4.77; *P*=.09) than the control group. Despite this, cardiac-related hospital admission costs were lower in the intervention group over 12 months (Aus $252.40 vs Aus $859.38; *P*=.24; a currency exchange rate of Aus $1=US $0.69 is applicable). There were no significant between-group differences in sedentary behavior minutes per day over 12 months, although the intervention group completed 22 minutes less than the control group (95% CI −22.80 to 66.69; *P*=.33; Cohen *d*=0.21). The intervention group had a lower BMI (β=1.62; *P*=.05), waist circumference (β=5.81; *P*=.01), waist-to-hip ratio (β=.03, *P*=.03), and quality of life (β=3.30; *P*=.05) than the control group. The intervention was more effective but more costly in reducing sedentary behavior (ICER Aus $351.77) and anxiety (ICER Aus $10,987.71) at 12 months. The intervention was also more effective yet costly in increasing quality of life (ICER Aus $93,395.50) at 12 months.

**Conclusions:**

The Vire app and ToDo-CR program was not an outcome-effective or cost-effective solution to reduce all-cause hospital admissions or ED presentations in cardiac rehabilitation compared with usual care. Smartphone apps that target sedentary behavior alone may not be an effective solution for cardiac rehabilitation participants to reduce hospital admissions and sedentary behavior.

**Trial Registration:**

Australian New Zealand Clinical Trials Registry (ANZCTR) ACTRN12619001223123; https://australianclinicaltrials.gov.au/anzctr/trial/ACTRN12619001223123

**International Registered Report Identifier (IRRID):**

RR2-10.1136/bmjopen-2020-040479

## Introduction

### Overview

Cardiovascular disease, including coronary heart disease (CHD), is the leading cause of death in Australia and globally [1-3]. Exercise-based cardiac rehabilitation for people with CHD is associated with significant risk reductions in cardiovascular mortality, hospitalizations, and repeat cardiac events [4]. Even so, 1 in 3 cardiac events are repeat events, and repeat cardiac events increase the risk of premature mortality [5].

### Cardiac Rehabilitation and Sedentary Behavior

Cardiac rehabilitation aims to reduce morbidity through positive lifestyle changes, including increasing physical activity and decreasing sedentary behavior [6,7]. Despite this, the sedentary behavior levels of cardiac rehabilitation participants remain high before, during, and after the program [8-16]. Sedentary behavior is associated with an increased risk of morbidity and all-cause mortality [17-21]. Accelerometry-measured sedentary times of ≥9 waking hours per day place healthy individuals at a significantly higher risk of death [18]. Television viewing times (a self-reported marker of sedentary behavior) in adults with diagnosed CHD or stroke of ≥4 hours per day is associated with a 52% increased risk of all-cause mortality compared with those watching television for ≤2 hours [21]. Breaking up sedentary time more frequently is also associated with decreased systolic blood pressure in cardiac rehabilitation participants [8]. Cardiac rehabilitation participants are likely to benefit by engaging in interventions that reduce sedentary behavior, and further options to support participants may be needed.

### Smartphone Apps to Reduce Hospital Admissions and Sedentary Behavior

Evidence suggests that cardiac rehabilitation participants are interested in support via the internet and mobile phones [22,23], and they have the potential to reduce hospital readmissions [24-26]. Widmer et al [25] reported that cardiac rehabilitation participants using a digital health intervention had significant (*P*=.04) reductions in weight and blood pressure and a 28% reduction in rehospitalizations and emergency department (ED) presentations compared with those who received traditional cardiac rehabilitation only. We hypothesized that better secondary prevention management of risk factors through cardiac rehabilitation plus the digital health intervention can lead to reduced hospital admissions. Similarly, smartphone apps that reduce sedentary behavior may be a feasible option to reduce hospital admissions. Few studies have targeted sedentary behavior change through smartphone apps in people with CHD [27-29]. These studies are generally small (≤50 participants), short in duration (≤3 mo), and aimed at examining the feasibility of such interventions [30]. Effect sizes have varied in these studies, with one reporting no change in self-reported sitting time [27], and in the feasibility trial preceding this study, a medium reduction in accelerometer-measured sedentary behaviors was reported [28]. One of the largest studies to date targeting sedentary behavior involving a smartphone app, pocket-worn activity tracker, and participants with CHD reported no significant between-group reduction in device-measured sedentary time [29]. However, cardiac rehabilitation participants using the SIT LESS protocol had reduced odds of sitting >9.5 hours per day [29]. Despite varying results, these studies concluded that smartphone apps may be feasible in reducing sedentary behavior in people with CHD and that larger-scale randomized controlled trials are warranted to determine their effectiveness. Therefore, we aimed to test the effectiveness of a sedentary behavior change smartphone app (Vire app and ToDo-CR program) as an adjunct to cardiac rehabilitation for hospital admissions and ED presentations over 12 months. As secondary aims, we examined the effectiveness of the Vire app and ToDo-CR program in decreasing accelerometer-measured sedentary behavior and cost-effectiveness.

## Methods

### Design

**Figure 1:**
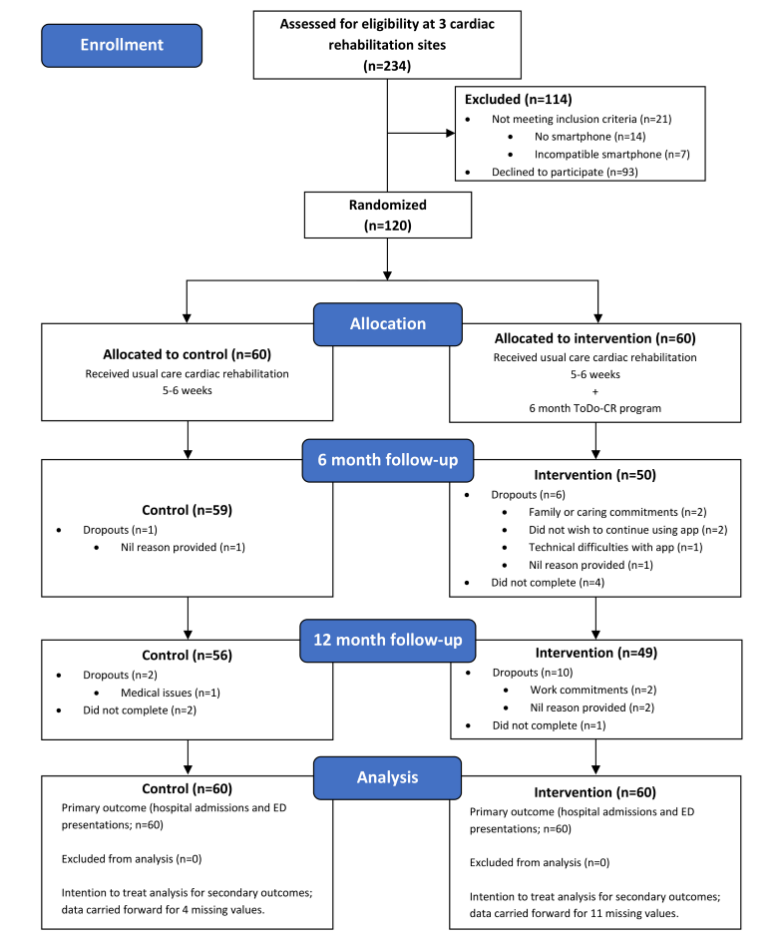
Flow of participants through the trial. ED: emergency department.

Using an assessor-blind, parallel randomized controlled trial design, participants were recruited from 3 phase-2 hospital-based cardiac rehabilitation programs in Canberra, Australia, between January 2020 and December 2021. The study duration for each participant was 12 months (Figure 1). Following the baseline assessment, participants were randomly assigned 1:1 to either usual care cardiac rehabilitation or the intervention: cardiac rehabilitation plus the 6-month behavior change smartphone app commencing at the start of cardiac rehabilitation (Vire app and ToDo-CR program) and followed up at 6 and 12 months at the University of Canberra by a blinded research assistant. The study protocol has been published elsewhere [31]. Participants were aged ≥18 years, had stable CHD, owned a smartphone, had no serious medical or functional impairments, and had adequate English language and cognitive skills [31].

### Ethical Considerations

All participants provided written informed consent. Ethics approval was obtained from the Australian Capital Territory Health (2019.ETH.00162), Calvary Public Hospital Bruce (20-2019), and the University of Canberra (HREC-2325) Human Research Ethics Committees. Reporting was informed by the CONSORT (Consolidated Standards of Reporting Trials)–EHEALTH [32] and CONSERVE statements [33] and guidelines for studies affected by the COVID-19 pandemic [34].

### Intervention

The Vire app and ToDo-CR behavior change program was informed by the *Do Something Different* approach focusing on breaking existing sedentary behavior habits and becoming behaviorally flexible [31,35,36]. The Vire app integrated data from a Fitbit Inspire wearable activity tracker provided to the participants and smartphone GPS data through machine learning to create a comprehensive digital profile of the participants’ current behavior. Using the data, the Vire app sends short, personalized behavior change messages known as *Do’s* in the form of push notifications 2 to 3 times per week at random over 6 months. The *Do’s* targeted sedentary behavior, suggesting microbehavioral alternatives designed to disrupt usual habits and encourage small lifestyle changes. Participants were encouraged to download the app, and technological support was provided throughout the trial.

### Outcome Measures

#### Nonelective Hospital Admissions and ED Presentations

The primary outcome was the total number of all-cause hospital admissions (nonelective admission to an acute hospital) and ED presentations within the 12 months after commencing cardiac rehabilitation. Data on cardiac-related hospital admissions and time frames to admission were also collected. Participants self-reported hospital admissions, which were verified by a comprehensive hospital patient records audit for all participants, regardless of self-report admission.

#### Sedentary Behavior and Other Secondary Outcomes

Sedentary behavior and physical activity were measured using a triaxial commercial accelerometer (ActiGraph wGT3X-BT) worn by participants on the right hip for 7 consecutive days during waking hours. ActiLife software (ActiLife V.6.13.4) was used to download raw data (30 Hz), which were converted to 15-second epochs and counts per minute (cpm) [9,16]. All data were screened and excluded if there was <10 hours per day wear time and if there were less than 4 days of valid data [9,16,37,38]. The following vector magnitude (VM) cut-off points were used: sedentary behavior, <150 cpm; light-intensity physical activity, 150 to 2689 cpm; and moderate to vigorous intensity physical activity, ≥2690 cpm [9,16,37,39,40].

Additional secondary outcomes included BMI (kg/m^2^); waist circumference; waist-to-hip ratio; blood pressure; exercise capacity (6-min walk test distance [41]); health-related quality of life (Assessment of Quality of Life [AQoL]-6D, a score of 100 reflects best health [42,43]); anxiety and depression (Hospital Anxiety and Depression Scale, a score of 0 reflects best outcomes [44]); and the stage of behavior change for physical activity (modified University of Rhode Island Change Assessment Scale-E2, scores of ≤8 indicate precontemplation, 9-11 indicate contemplation, and 12-14 indicate action and maintenance [45]).

#### Smartphone App Usability and Engagement

For those in the intervention group, the usability and acceptance of the Vire app and ToDo-CR program were assessed using the Unified Theory of Acceptance and Use of Technology questionnaire [46]. Engagement with the Vire app was assessed by viewing app logs showing the completion of *Do’s*. The total number of completed *Do’s* across the study was recorded.

#### Economic Evaluation

The costs of implementing and delivering the intervention were recorded prospectively, including payment for the Vire app and maintenance of the server, the purchase of Fitbit Inspire wearable activity trackers, and phone call and email support related to the app from a cardiac rehabilitation clinician.

A hospital patient records audit was completed for all participants to obtain the associated Australian Refined Diagnosis Related Group (AR-DRG) classification code for any nonelective admissions and the Urgent Related Group classification codes for any ED presentation. The Independent Health and Aged Care Pricing Authority Australian version of the National Weighted Activity Unit calculators were used to obtain hospital cost information [47,48]. Costs are reported in Aus $ throughout. A currency exchange rate of Aus $1=US $0.69 is applicable

### Sample Size Calculation

Sample size was based on detecting a significant difference in hospital admissions and ED presentations between usual care and a digital health intervention. A similar study [25] noted a 28% difference in rehospitalization and ED presentations between usual care cardiac rehabilitation (a standard rate of 44%) and cardiac rehabilitation plus a digital health intervention (a standard rate of 16%). Using a 2-sided significance of *P*<.05 and power of 88% (calculated using G*Power 3.1.9.4), a minimum of 108 participants were needed. Accounting for a 25% dropout rate, 72 participants per group (144 total sample = 108 / [1 – 0.25]) were calculated.

### Changes in Response to the COVID-19 Pandemic

To comply with public health recommendations, there were variations in the methods used in the protocol [31]. The trial was extended by 12 months owing to the closure of the cardiac rehabilitation programs. The required sample size was also reduced from 144 to a minimum of 108 (removing the 30% dropout buffer) owing to the impact of the COVID-19 pandemic [31]. In addition, all participants experienced varying restrictions and closures of nonessential health care services. Due to COVID-19 pandemic restrictions, some follow-up assessments were completed via telehealth (Zoom video call). Participants used their own blood pressure monitors, scales, and tape measures under the instruction of a blinded assessor. The method of assessment was documented throughout the study. All changes were approved by the overseeing ethics review committees.

### Analysis

Data were analyzed according to group assignment using intention-to-treat analyses. Missing data were handled by bringing the last value forward (carryover approach). An on-protocol analysis was also performed. All descriptive statistics were reported using means and SDs, medians, and IQRs or proportions, as appropriate. Normality was assessed using the Kolmogorov-Smirnov test for samples ≥50.

The primary analysis was the comparison of rates of nonelective hospital admissions and ED presentations. Binary logistic regression for “yes” versus “no” admissions (odds ratios) and negative binomial with log link regression for the rate or number of admissions (incidence rate ratio) were completed. Survival analyses (Cox regression) were completed to consider the time frame to admission (hazard ratios). Adjustments were made for sociodemographic variables (eg, age and sex) and other covariates (eg, diabetes and other chronic diseases).

Linear mixed-effects models for repeated measures were used to analyze all other secondary outcomes. The maximum likelihood method was used for parameter estimation. Time and between-group comparisons were explored as fixed effects while adjusting for demographic characteristics (eg, age and sex) and other covariates (eg, education, employment, and accelerometer counts/d) [49-51]. Participants were treated as random effects (ie, random intercept models), and the intraclass correlation coefficient was reported. The model with best fit was informed by the Akaike Information Criteria [52]. All estimated effects (β) are reported with their associated 95% CIs. The correlation between outcomes and engagement with the Vire app and ToDo-CR program was also explored.

Sensitivity analyses were completed for outcomes self-administered by participants under telehealth conditions (eg, waist circumference and blood pressure) and for participants wearing their accelerometer belt during COVID-19 pandemic lockdowns and restrictions. Subanalyses were completed for participants excluded versus participants consented, dropouts versus those who completed the study, and those with prior experience with a physical activity tracker versus those with no experience. Data were analyzed using SPSS (version 27; IBM Corp).

### Cost-Effectiveness Analysis

Effectiveness was measured in terms of the secondary outcomes. The incremental cost-effectiveness ratios (ICERs) were calculated using the following formula:

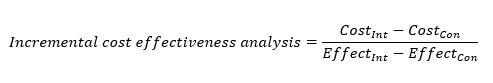



The mean difference in health care resource use costs included the costs of implementing the intervention plus the indirect costs associated with health care use (eg, hospital admissions and ED presentations). The difference in effects was calculated as the change from baseline to 6 months and from baseline to 12 months for each secondary outcome per individual participant. The mean difference of these individual differences was then calculated using 2-tailed independent samples *t* tests with 95% CIs. From these analyses, the additional cost per unit of health benefit gained (by using the Vire app and ToDo-CR program compared with usual care alone) was determined.

## Results

### Overview

A total of 120 participants were recruited for this trial (Figure 1). Participant characteristics are reported in Table 1. The majority were male (93/120, 77.5%), tertiary educated (95/120, 79.2%), and employed (62/120, 51.7%). Approximately half (55/120, 45.8%) of the participants had prior experience using physical activity tracking apps or wearable trackers. The participants who were assessed for eligibility and excluded (n=114) were significantly older (67 vs 63 y; *P*=.003), from public hospitals (*P*=.001), and less likely to have had a percutaneous coronary intervention and more likely to have had a myocardial infarction (*P*=.01; Table S1 in Multimedia Appendix 1). The main reason for exclusion was declining to participate, with the primary reason for declining being “not interested in smartphone apps” (28/93, 30%). The only reasons for not meeting the inclusion criteria were not having a smartphone (14/21, 67%) and having an incompatible smartphone (7/21, 33%; Table S2 in Multimedia Appendix 1). Retention rates were high in both groups (intervention 49/60, 82% vs control 56/60, 93%); however, there was a significant difference between the 2 groups’ retention rates (*P*=.02) but no difference in characteristics (Table S3 in Multimedia Appendix 1).

**Table 1:** Characteristics of participants at baseline.

Characteristics			Control (n=60)		Intervention (n=60)		Total (N=120)
Age (years), mean (SD)			64.10 (9.99)		61.12 (10.06)		62.61 (10.10)
Sex (male), n (%)			48 (80)		45 (75)		93 (77.5)
Country of birth (Australia), n (%)			39 (65)		40 (66.7)		79 (65.8)
**Education, n (%)**							
	Secondary	10 (16.7)		15 (25)		25 (20.8)	
	Tertiary	50 (83.3)		45 (75)		95 (79.2)	
**Employment, n (%)**							
	Full time	23 (38.3)		25 (41.7)		48 (40)	
	Part time	4 (6.7)		10 (16.7)		14 (11.7)	
	Voluntary work	4 (6.7)		1 (1.7)		5 (4.2)	
	Not in the labor force	29 (48.3)		24 (40)		53 (44.2)	
Relationship status (partner), n (%)			55 (91.7)		48 (80)		103 (85.8)
**Previous experience with physical activity trackers, n (%)**							
	Smartphone app	8 (13.3)		13 (21.7)		21 (17.5)	
	Smartwatch	11 (18.3)		12 (20)		23 (19.2)	
	Both	6 (10)		5 (8.3)		11 (9.2)	
	Neither	35 (58.3)		30 (50)		65 (54.2)	
**Diagnosis, n (%)**							
	Stable coronary heart disease	1 (1.7)		2 (3.3)		3 (2.5)	
	CABG^a^	12 (20)		12 (20)		24 (20)	
	PCI^b^	32 (53.3)		27 (45)		59 (49.2)	
	Myocardial infarction	1 (1.7)		2 (3.3)		3 (2.5)	
	Myocardial infarction + PCI	14 (23.3)		17 (28.3)		31 (25.8)	
Type 2 diabetes (yes), n (%)			12 (20)		13 (22)		25 (20.8)
Other chronic disease (yes), n (%)			10 (16.7)		21 (35)		31 (25.8)
Blood pressure medication (yes), n (%)			50 (83.3)		46 (76.7)		97 (80.8)
Cholesterol medication (yes), n (%)			57 (95)		58 (96.7)		115 (95.8)
Other cardiac medications (yes), n (%)			58 (96.7)		55 (91.7)		113 (94.2)
Current smoker (yes), n (%)			1 (1.7)		2 (3.3)		3 (2.5)
**Cardiac rehabilitation system, n (%)**							
	Public	37 (61.7)		38 (63.3)		75 (62.5)	
	Private	23 (38.3)		22 (36.7)		45 (37.5)	
**Cardiac rehabilitation model, n (%)**							
	Face-to-face	51 (85)		53 (88.3)		104 (86.7)	
	Hybrid	2 (3.3)		2 (3.3)		4 (3.3)	
	Telehealth	7 (11.7)		5 (8.3)		12 (10)	
Cardiac rehabilitation sessions attended (%), mean (SD)			86 (24)		83 (28)		84 (26)
Previous attendance to cardiac rehabilitation (yes), n (%)			7 (11.7)		9 (15)		16 (13.3)

^a^CABG: coronary artery bypass graft.

^b^PCI: percutaneous coronary intervention.

### Nonelective Hospital Admissions and ED Presentations

Nonelective hospital admissions and ED presentations for all participants are shown in Table 2. The most frequent cause of admission was chest pain (AR-DRG code F74B). The results of the logistic regression models on the likelihood that participants have a nonelective hospital admission or ED presentation are reported in Table 3. After adjustment for age, sex, and the presence of diabetes and other chronic diseases, those in the intervention group were 1.54 times more likely to have an admission, 3.26 times more likely to have a cardiac-related admission, and 2.07 times more likely to have an ED presentation than those in the control group.

Group allocation was not a significant predictor of the incidence (number) of all-cause or cardiac-related admissions or ED presentations (Table 4). The admission rate among participants in the intervention group was 1.69 times higher than the rate in the control group for all-cause admission, 1.56 times higher for cardiac-related admissions, and 1.90 times higher for ED presentations following adjustment for age, sex, and the diagnosis of diabetes or other chronic diseases (Table 4).

Results of the Cox regression analysis to determine if there were differences in time to admission or ED presentation between the intervention and control groups are presented in Table S4 in Multimedia Appendix 1. The intervention group participants had 1.52 times the probability of experiencing an all-cause admission in 12 months compared with the control group. They also had 3.14 times the probability of experiencing a cardiac-related admission and 1.84 times the probability of experiencing an ED presentation when adjusted for age, sex, and diagnosis of diabetes and other chronic diseases (Table S4 in Multimedia Appendix 1).

**Table 2:** Nonelective hospital admissions and emergency department presentations within 12 months of commencing cardiac rehabilitation.

		Control (n=60)	Intervention (n=60)	Difference between groups (intervention-control)	*P* value
**Nonelective hospital admissions**					
	Proportion of participants who had at least one hospital admission (yes), n (%)	10 (17)	12 (20)	2 (3)	.64
	Total number of admissions, n	13	18	5	.48
	Cardiac-related admissions^a^, n (%)	6 (46)	12 (67)	6 (10)	.22
	Time frame from cardiac rehabilitation to admission (d), mean (SD)	131.50 (86.85)	117.25 (94.30)	−14.25 (81.30)	.83
**Emergency department presentations**					
	Proportion who had an emergency department presentation (yes), n (%)	15 (25)	22 (37)	7 (12)	.17
	Total number of emergency department presentations, n	20	36	16	.10

^a^Cardiac-related admission determined using Australian Refined Diagnosis Related Group codes (F01A to F10B, F12A to F12B, F14A to F19B, F22Z to F60B, F66A to F67B, and F69A to F76B).

**Table 3:** The odds of nonelective hospital admissions and emergency department presentations within 12 months using logistic regression models.

Dependent variable^a^	Model 1^b^			Model 2^c^			Model 3^d^	
	OR^e^ (95% CI)	*P* value	OR (95% CI)		*P* value	OR (95% CI)		*P* value
Nonelective all-cause hospital admissions	1.24 (0.49-3.16)	.64	1.31 (0.51-3.38)		.58	1.54 (0.58-4.10)		.39
Nonelective cardiac-related hospital admissions^f^	2.15 (0.617.58)	.23	2.24 (0.63-8.00)		.22	3.26 (0.84-12.55)		.09
Emergency department presentations	1.74 (0.79-3.81)	.17	1.79 (0.80-3.98)		.16	2.07 (0.89-4.77)		.09

^a^Reference=control.

^b^Model 1: nil adjustments.

^c^Model 2: adjusted for age and sex.

^d^Model 3: adjusted for age, sex, diabetes, and presence of other chronic diseases.

^e^OR: odds ratio.

^f^Cardiac-related admission determined using Australian Refined Diagnosis Related Group codes (F01A to F10B, F12A to F12B, F14A to F19B, F22Z to F60B, F66A to F67B, and F69A to F76B).

**Table 4:** The incidence of nonelective hospital admissions and emergency department presentations within 12 months using negative binomial regression with log link.

Dependent variable^a^	Model 1^b^			Model 2^c^			Model 3^d^	
	IRR^e^ (95% CI)	*P* value	IRR (95% CI)		*P* value	IRR (95% CI)		*P* value
Nonelective all-cause hospital admissions	1.39 (0.62-3.08)	.42	1.46 (0.65-3.31)		.36	1.69 (0.73-3.92)		.23
Nonelective cardiac-related hospital admissions^f^	1.83 (0.51-6.57)	.35	1.76 (0.46-6.73)		.41	1.56 (0.38-6.48)		.54
Emergency department presentations	1.80 (0.94-3.46)	.08	1.78 (0.91-3.44)		.09	1.90 (0.95-3.79)		.07

^a^Reference=control.

^b^Model 1: nil adjustments.

^c^Model 2: adjusted for age and sex.

^d^Model 3: adjusted for age, sex, diabetes, and the presence of other chronic diseases.

^e^IRR: incidence rate ratio.

^f^Cardiac-related admission determined using Australian Refined Diagnosis Related Group codes (F01A to F10B, F12A to F12B, F14A to F19B, F22Z to F60B, F66A to F67B, and F69A to F76B).

### Secondary Outcomes

Sedentary behavior and physical activity measures are reported in Tables 5 and 6 and Table S5 in Multimedia Appendix 1. Both the intervention and control groups showed an increase in sedentary behavior over 12 months, spending approximately 10 hours per day in sedentary behaviors (Table S5 in Multimedia Appendix 1). There were no significant between- or within-group differences in any sedentary behavior measures, although there was a small effect size for the reduction in sedentary behavior (min/d) at 6 (Cohen *d*=0.11) and 12 months (Cohen *d*=0.21) in favor of the intervention group compared with the control group. At 6 and 12 months, the control group engaged in 15 minutes (*P*=.54) and 22 minutes (*P*=.33) more sedentary behavior than the intervention group when adjusted for age, sex, VM counts, employment, and education (Tables 5 and 6). In subanalyses, those in the intervention group who had prior experience with physical activity trackers (n=30) spent a lower percentage of the day in sedentary behavior (mean difference 6.13%, 95% CI 0.97%-11.28%; *P*=.02), had shorter sedentary bouts (mean difference 2.32 min, 95% CI 0.36-4.27; *P*=.02), and had lower overall sedentary minutes per day (mean difference 65.22 min, 95% CI −13.29 to 143.72; *P*=.10) at 6 months on the completion of the intervention (Table S6 in Multimedia Appendix 1). There were no significant differences in the control group based on prior physical activity tracker use (Table S7 in Multimedia Appendix 1).

There were no significant between-group differences at 6 or 12 months in any physical activity measures, except for wear time at 12 months (Table S5 in Multimedia Appendix 1). Nonetheless, the control group showed a significant within-group increase in VM counts at 6 months and light-intensity physical activity at 12 months. The results of the linear mixed-effects models are reported in Tables 5 and 6, and all models were nonsignificant.

All other secondary outcomes are presented in Table S8 in Multimedia Appendix 1. The control group had a higher BMI (Cohen *d*=0.30), waist circumference (Cohen *d*=0.43), and waist-to-hip ratio (Cohen *d*=0.33) at 6 and 12 months (Table S8 in Multimedia Appendix 1). Using linear mixed-effects models, BMI, waist circumference, and waist-to-hip ratio remained higher in the control group after adjustment for age, sex, employment, and education (Table S9 in Multimedia Appendix 1).

There was no significant between-group difference for systolic and diastolic blood pressure, 6-minute walking distance, anxiety, or depression (Tables S8 and S9 in Multimedia Appendix 1). There was a significant between-group difference in favor of the control group for quality of life (Cohen *d*=0.14; *P*=.03*;* Table S8 in Multimedia Appendix 1). In linear mixed models, the control group experienced a significantly higher overall quality of life at 6 and 12 months when adjusted for age, sex, employment, and education (Table S9 in Multimedia Appendix 1).

On the basis of the University of Rhode Island Change Assessment Scale readiness to change scores, participants were in the precontemplation stage throughout the study regarding physical activity change. There were no significant within-group or between-group differences (Table S8 in Multimedia Appendix 1) or significant differences over time (Table S9 in Multimedia Appendix 1). No significant differences were observed in sensitivity analyses for secondary outcomes between those wearing accelerometers during the COVID-19 pandemic lockdowns or completing telehealth measures.

**Table 5:** Difference in sedentary behavior and physical activity outcomes over 6 months between groups using linear mixed-effects models.

Dependent variable^a^	Model 1^b^				Model 2^c^				Model 3^d^		
	β (95% CI)	*P* value	ICC^e^	β (95% CI)		*P* value	ICC	β (95% CI)		*P* value	ICC
SB^f^ (min/d)	18.92 (−32.16 to 70.00)	.47	0.71	18.62 (−29.13 to 66.36)		.44	0.66	14.91 (−32.85 to 62.67)		.54	0.65
Percentage of SB/d (SB/wear time)	1.21 (−2.12 to 4.53)	.47	0.75	.77 (−1.20 to 2.75)		.44	0.63	.63 (−1.34 to 2.61)		.53	0.63
Average duration of SB bouts (min)	−.50 (−1.65 to 0.66)	.40	0.62	−.90 (−1.91 to 0.12)		.08	0.55	−.93 (−1.94 to 0.08)		.07	0.54
Number of SB bouts/d	.88 (−1.08 to 2.83)	.38	0.72	.66 (−1.08 to 2.39)		.46	0.66	.50 (−1.23 to 2.23)		.57	0.65
Number of SB breaks/d	.87 (−1.08 to 2.82)	.38	0.72	.66 (−1.08 to 2.39)		.46	0.66	.50 (−1.23 to 2.23)		.57	0.65
MVPA^g^ (min/d)	1.01 (−9.62 to 11.64)	.85	0.72	3.48 (−0.47 to 7.42)		.08	0.72	3.45 (−0.52 to 7.42)		.09	0.72
LPA^h^ (min/d)	−11.59 (−35.96 to 12.78)	.35	0.70	−8.98 (−27.20 to 9.24)		.33	0.66	−8.44 (−26.74 to 9.86)		.36	0.66
Steps/d	−284.79 (−1323.94 to 754.36)	.59	0.69	−142.76 (−620.86 to 335.34)		.56	0.71	−146.87 (−628.19 to 334.45)		.55	0.71

^a^Reference=intervention.

^b^Model 1: nil adjustments.

^c^Model 2: adjusted for age and sex.

^d^Model 3: adjusted for age, sex, diabetes, and the presence of other chronic diseases.

^e^ICC: intraclass correlation coefficient.

^f^SB: sedentary behavior.

^g^MVPA: moderate to vigorous intensity physical activity.

^h^LPA: light-intensity physical activity.

**Table 6:** Difference in sedentary behavior and physical activity outcomes over 12 months between groups using linear mixed-effects models.

Dependent variable^a^	Model 1^b^				Model 2^c^				Model 3^d^		
	β (95% CI)	*P* value	ICC^e^	β (95% CI)		*P* value	ICC^e^	β (95% CI)		*P* value	ICC^e^
SB^f^ (min/d)	24.52 (−24.15 to 73.20)	.32	0.66	24.50 (−20.08 to 69.09)		.28	0.62	21.94 (−22.80 to 66.69)		.33	0.62
Percentage of SB/d (SB/wear time)	1.31 (−2.01 to 4.62)	.44	0.74	.98 (−0.92 to 2.86)		.31	0.67	.85 (−1.04 to 2.74)		.38	0.67
Average duration of SB bouts (min)	−.19 (−1.42 to 1.04)	.76	0.69	−.57 (−1.68 to 0.54)		.31	0.63	−.62 (−1.72 to 0.49)		.27	0.63
Number of SB bouts/d	.90 (−0.95 to 2.75)	.33	0.65	.75 (−0.85 to 2.35)		.36	0.59	.64 (−0.95 to 2.24)		.43	0.59
Number of SB breaks/d	.89 (−0.95 to 2.74)	.34	0.65	.75 (−0.85 to 2.34)		.36	0.59	.64 (−0.96 to 2.24)		.43	0.58
MVPA^g^ (min/d)	1.42 (−9.04 to 11.87)	.79	0.73	3.10 (−0.80 to 6.99)		.12	0.73	3.06 (−0.86 to 6.98)		.13	0.73
LPA^h^ (min/d)	−9.48 (−33.96 to 14.99)	.44	0.73	−7.66 (−25.31 to 9.98)		.39	0.72	−7.01 (−24.66 to 10.65)		.43	0.72
Steps/d	−138.84 (−1151.26 to 873.58)	.79	0.74	−37.65 (−508.40 to 433.10)		.87	0.73	−36.24 (−510.30 to 437.83)		.88	0.73

^a^Reference=intervention.

^b^Model 1: nil adjustments.

^c^Model 2: adjusted for age and sex.

^d^Model 3: adjusted for age, sex, diabetes, and the presence of other chronic diseases.

^e^ICC: intraclass correlation coefficient.

^f^SB: sedentary behavior.

^g^MVPA: moderate to vigorous intensity physical activity.

^h^LPA: light-intensity physical activity.

### Engagement and Usability of the Vire App

The median completion rate of *Dos* was 3 of 55 (IQR 0-37.75) over 6 months. There were 27% (16/60) of participants who engaged with the app for the entire 6 months (ie, completed at least 1 *Do*/mo), 33% (20/60) of participants who engaged with the app less than once per month, and 40% (24/60) of participants who did not complete any *Dos*. In logistic regression modeling, those who had used apps or wearable activity trackers before were 1.04 times (*P*=.01) more likely to complete *Dos* (χ^2^_1_=7.1). Those who were employed full time or part time were 0.97 times (*P*=.01) less likely to complete *Dos* (χ^2^_1_=6.6). There were no statistically significant correlations among the total number of *Dos* completed and age (*r*=−0.07; *P*=.60), sedentary behavior (*r*=−0.15; *P*=.25), physical activity (*r*=0.21; *P*=.11), quality of life (*r*=0.18; *P*=.17), anxiety (*r*=−0.12; *P*=.35) or depression (*r*=−0.15; *P*=.26) at 6 months (Table S10 in Multimedia Appendix 1).

The usability of the Vire app (Unified Theory of Acceptance and Use of Technology) is presented in Table S11 in Multimedia Appendix 1. Participants were relatively satisfied with the usability of the Vire app, with a median score of ≥4 in all constructs except for habit (score=3.54/7) and use (score=3.29/7).

### Economic Evaluation

#### Overview

On average, the cost of implementing the intervention per participant was Aus $1086.55 (Table S12 in Multimedia Appendix 1). The mean cost of all-cause and cardiac-related hospital admissions was higher in the control group at 6 months and remained higher in the control group for cardiac-related admissions at 12 months. However, these costs were nonsignificant between groups (Table S13 in Multimedia Appendix 1). The health care use costs for ED presentations at 6 months were significantly higher in the intervention group (Aus $315.83 vs Aus $136.00; *P*=.04).

#### Cost-Effectiveness

The cost-effectiveness of the Vire app and ToDo-CR program compared with usual care is presented in Table 7 for the secondary outcomes. Although the intervention was costlier to implement and this group had higher health care use, it was also more effective at reducing the BMI, increasing light-intensity physical activity at 6 months, improving quality of life and anxiety symptoms at 6 and 12 months, and reducing sedentary behavior at 12 months. The ICERs presented in Table 6 represent the cost per unit change for each outcome. For example, the cost of a 1-minute reduction in sedentary time at 12 months was Aus $351.77.

**Table 7:** Health care use cost and health benefit differences at 6 and 12 months considering all-cause and cardiac-related nonelective hospital admissions per participant.

				All cause									Cardiac related^a^						
				Intervention, mean change (SD)		Control, mean change (SD)		Mean difference (95% CI)		ICER^b^ (Aus $^c^)		Intervention, mean change (SD)			Control, mean change (SD)		Mean difference (95% CI)		ICER (Aus $)
**Baseline to 6 mo**																			
	**Health care use costs (Aus $)^d^**		2041.53 (1860.55)^e^		1423.63 (6248.96)		617.90 (−1048.97 to 2284.77)		—^f^		1819.23 (1246.59)			1079.65 (6096.69)		739.58 (−851.30 to 2330.46)		—	
		Hospital admissions	361.00 (1287.94)		1111.48 (5948.69)		−750.48 (−2306.51 to 805.55)		—		138.70 (413.12)			767.50 (5838.20)		−628.80 (−2125.08 to 867.48)		—	
		ED^g^	315.83 (605.23)		136.00 (326.61)		179.83 (3.46 to 356.20)		—		—			—		—		—	
	**Effectiveness**																		
		SB^h^ (min/d)	16.84 (109.94)		14.43 (114.34)		2.41 (−38.15 to 42.96)		256.39		16.84 (109.94)			14.43 (114.34)		2.41 (−38.15 to 42.96)		306.88	
		MVPA^i^ (min/d)	0.37 (24.20)		4.65 (20.02)		−4.28 (−12.31 to 3.75)		−144.37		0.37 (24.20)			4.65 (20.02)		−4.28 (−12.31 to 3.75)		−172.80	
		LPA^j^ (min/d)	11.80 (60.92)		10.84 (47.55)		0.95 (−18.80 to 20.71)		643.65		11.80 (60.92)			10.84 (47.55)		0.95 (−18.80 to 20.71)		770.40	
		BMI (kg/m^2^)	−0.20 (1.29)		0.03 (1.35)		−0.23 (−0.71 to 0.25)		−2686.52		−0.20 (1.29)			0.03 (1.35)		−0.23 (−0.71 to 0.25)		−3215.57	
		Waist circumference (cm)	−1.29 (4.50)		−2.18 (6.23)		0.89 (−1.08 to 2.86)		694.27		−1.29 (4.50)			−2.18 (6.23)		0.89 (−1.08 to 2.86)		830.99	
		Systolic blood pressure (mm Hg)	5.52 (12.87)		3.36 (14.81)		2.16 (−3.41 to 7.74)		286.06		5.52 (12.87)			3.36 (14.81)		2.16 (−3.41 to 7.74)		342.40	
		6-min walk test distance (m)	55.69 (62.19)		84.42 (127.01)		−28.73 (−77.89 to 20.42)		−21.51		55.69 (62.19)			84.42 (127.01)		−28.73 (−77.89 to 20.42)		−25.74	
		AQoL^k^-6D Utility (0-1.0)	0.03 (0.10)		0.02 (0.07)		0.004 (−0.03 to 0.04)		61,790.00		0.03 (0.10)			0.02 (0.07)		0.004 (−0.03 to 0.04)		73,958.00	
		Anxiety (0-21)	−0.57 (2.13)		−0.35 (2.07)		0.38 (−0.97 to 0.54)		−2808.64		−0.57 (2.13)			−0.35 (2.07)		−0.22 (−0.97 to 0.54)		−3361.73	
		Depression (0-21)	−0.45 (1.67)		−0.58 (1.92)		0.13 (−0.52 to 0.78)		4753.08		−0.45 (1.67)			−0.58 (1.92)		0.13 (−0.52 to 0.78)		5689.08	
**Baseline to 12 mo**																			
	**Health care use costs (Aus $)^c^**		3507.08 (11,730.49)		1639.17 (6359.44)		1867.92 (−1543.35 to 5279.19)		—		1932.93 (1640.97)			1171.53 (6134.12)		761.40 (−861.94 to 2384.74)		—	
		Hospital admissions	1826.55 (11,454.49)		1327.02 (6007.08)		499.53 (−2807.09 to 3806.16)		—		252.40 (784.37)			859.38 (5850.84)		−606.98 (−2116.14 to 092.18)		—	
		ED	593.98 (1046.12)		312.15 (686.72)		281.83 (−38.61 to 602.28)		—		—			—		—		—	
	**Effectiveness**																		
		SB (min/d)	19.99 (105.30)		25.30 (156.05)		−5.31 (−53.44 to 42.82)		−351.77		19.99 (105.30)			25.30 (156.05)		−5.31 (−53.44 to 42.82)		−143.39	
		MVPA (min/d)	−1.39 (29.49)		1.94 (19.64)		−3.33 (−12.39 to 5.72)		−9.56		−1.39 (29.49)			1.94 (19.64)		−3.33 (−12.39 to 5.72)		−228.65	
		LPA (min/d)	7.16 (64.21)		13.80 (52.34)		−6.64 (−27.82 to 14.54)		−281.31		7.16 (64.21)			13.80 (52.34)		−6.64 (−27.82 to 14.54)		−114.67	
		BMI (kg/m^2^)	0.38 (1.34)		0.34 (1.44)		0.04 (−0.46 to 0.55)		46,697.75		0.38 (1.34)			0.34 (1.44)		0.04 (−0.46 to 0.55)		19,035.00	
		Waist circumference (cm)	0.37 (5.64)		−0.42 (5.61)		0.78 (−1.25 to 2.82)		2364.44		0.37 (5.64)			−0.42 (5.61)		0.78 (−1.25 to 2.82)		963.80	
		Systolic blood pressure (mm Hg)	4.59 (13.12)		7.36 (20.81)		−2.77 (−9.84 to 4.29)		−674.34		4.59 (13.12)			7.36 (20.81)		−2.77 (−9.84 to 4.29)		−274.87	
		6-min walk test distance (m)	96.95 (130.96)		97.37 (129.12)		−0.43 (−59.09 to 58.23)		−4344.00		96.95 (130.96)			97.37 (129.12)		−0.43 (−59.09 to 58.23)		−1770.70	
		AQoL-6D Utility (0-1.0)	0.04 (0.09)		0.02 (0.12)		0.02 (−0.02 to 0.06)		93,395.50		0.04 (0.09)			0.02 (0.12)		0.02 (−0.02 to 0.06)		38,070.00	
		Anxiety (0-21)	−0.65 (1.87)		−0.48 (2.04)		−0.17 (−0.87 to 0.54)		−10,987.71		−0.65 (1.87)			−0.48 (2.04)		−0.17 (−0.87 to 0.54)		−4478.82	
		Depression (0-21)	−0.35 (1.80)		−0.95 (2.03)		0.60 (−0.09 to 1.29)		3113.18		−0.35 (1.80)			−0.95 (2.03)		0.60 (−0.09 to 1.29)		1269.00	

^a^Cardiac-related admission determined by Australian Refined Diagnosis Related Group codes (F01A to F10B, F12A to F12B, F14A to F19B, F22Z to F60B, F66A-F67B, and F69A to F76B).

^b^ICER: incremental cost-effectiveness ratio.

^c^A currency exchange rate of Aus $1=US $0.69 is applicable.

^d^Direct cost of intervention and indirect cost of either all-cause or cardiac-related hospital admissions and emergency department presentations per participant, Aus $.

^e^Includes cost of intervention: Aus $1086.55. ICER = (intervention cost – control cost) / (intervention effect – control effect), where effect is the health outcome.

^f^Health care use costs are accounted for in the ICER calculation for secondary outcomes.

^g^ED: emergency department.

^h^SB: sedentary behavior.

^i^MVPA: moderate to vigorous intensity physical activity.

^j^LPA: light-intensity physical activity.

^k^AQoL: Assessment of Quality of Life.

## Discussion

### Principal Findings

The use of the Vire app and ToDo-CR program was not effective in reducing hospital admissions and ED presentations nor did it significantly decrease sedentary behavior compared with usual care over 12 months. Participants in the intervention group were more likely to have a cardiac-related hospital admission; however, the costs of these admissions were markedly lower than those in the control group. Although the intervention was costlier to implement, it was also more effective at reducing sedentary behavior, BMI, and anxiety and increasing quality of life and light-intensity physical activity. Retention rates were high in this study; however, engagement with the Vire app and ToDo-CR program was low. Furthermore, there was no correlation between age and engagement with the Vire app and ToDo-CR program; instead, there was a correlation between prior experience with physical activity trackers and apps.

### Comparison With Prior Work

Intervention participants were 50% more likely to have a hospital admission, twice as likely to have an ED presentation, and 3 times more likely to be admitted to the hospital for a cardiac-related hospital admission. This contrasts with the studies by Widmer et al [25] and Rivers et al [26], who each noted an approximate 30% decrease in the rate of hospital admissions in the groups using an app-based intervention in cardiac rehabilitation. These studies targeted a range of lifestyle risk factors (eg, physical activity, smoking, diet, and medication adherence) rather than 1 risk factor. Perhaps by targeting only sedentary behavior, the strength of the intervention was not enough to produce significant changes in the primary outcome, noting that multiple factors contribute to hospital admissions and risk factor control such as medication and comorbidity management [53].

Cardiac rehabilitation is associated with significant reductions in hospitalizations and repeat cardiac events [4]. Nonetheless, of those who are referred for cardiac rehabilitation in Australia, only 28% attend [54]. Alternate technology-based methods (such as the Vire app and ToDo-CR program) may therefore be better suited to reach those not attending traditional cardiac rehabilitation who are at a higher risk of hospitalizations compared with those who attend traditional cardiac rehabilitation. This group is missing the behavior change advice and support provided in cardiac rehabilitation and may have the most to gain by engaging. Providing the option of an app-based cardiac rehabilitation program is associated with increased overall cardiac rehabilitation participation rates [26] by removing barriers such as the need to travel. In addition, app-based cardiac rehabilitation programs have been shown to achieve outcomes comparable with traditional programs [24,55,56]. One study reported readmission data comparing no cardiac rehabilitation, traditional cardiac rehabilitation, and app-based cardiac rehabilitation [26]. Although this study was not designed to evaluate differences in readmissions, cardiac-related admissions were lower in those using the app (1/23, 4%) versus those receiving no cardiac rehabilitation (5/39, 13%) [26]. Larger-scale studies are required to investigate the effect of smartphone apps in those declining cardiac rehabilitation and the possible benefits to hospitalizations.

Those in the intervention group attended the ED more frequently than those in the control group, and the most frequent cause for admission was chest pain (AR-DRG code F74B). Presenting to the ED for chest pain is particularly relevant with advice given in the Vire app and ToDo-CR program to seek medical attention if participants experienced these symptoms. These participants may have become more proactive in self-managing their CHD because of the education provided. Public education campaigns to raise awareness of the signs and symptoms of acute chest pain have been shown to be effective in increasing the rate of ED presentations for early medical intervention [57,58]. Despite having more admissions, the intervention group costs associated with cardiac-related admissions were lower (intervention, Aus $252.40 vs control, Aus $859.30), meaning that when they were admitted, they potentially had shorter length of stays for less-severe diagnoses, requiring less-costly medical intervention and overall less impact on the health care system. Delays in presenting to the ED with acute chest pain, a potential indicator of a myocardial infarction, contribute to patient morbidity and mortality [57,59]. Smartphone apps are being developed to support patients with CHD and incorporate educational features regarding symptoms of chest pain, with a key focus being to support participants rather than just monitor them [60]. These types of features and providing education may be especially important in future studies for those not attending face-to-face cardiac rehabilitation programs.

Although the effect size for reduction in sedentary behavior was small in this study, an approximate 20-minute reduction may still be clinically meaningful. In the limited studies available that test the effect of smartphone apps on sedentary behavior [30], greater reductions have been seen compared with this study in cardiac rehabilitation participants (accelerometer-measured 100-min reduction/d at 4 mo [28]; accelerometer-measured 96-min reduction/d at 3 mo [29]); stroke (accelerometer-measured 60-min reduction/d at 6 wk [61]); and chronic stroke (self-reported 180-min reduction/d at 3 mo [62]). There is no well-established, minimal clinically important difference for sedentary behavior in people with cardiovascular disease including CHD. Studies in the general population have reported that for every 30 minutes of sedentary behavior reallocated to light-intensity or moderate to vigorous intensity physical activity, there is a 2% to 25% improvement in cardiovascular disease risk biomarkers [63], and a 1- to 2-hour reduction in television viewing time is associated with reductions in cardiovascular disease risk (eg, waist circumference, BMI, triglycerides, insulin sensitivity, and blood pressure) [64,65]. In addition, a break in sedentary behavior as short as 1 minute can reduce waist circumference and improve C-reactive protein (inflammatory marker) independent of total sedentary time [65,66]. This study showed minimal change in sedentary bouts and breaks between groups or over time. Sedentary behavior bouts and breaks are also infrequently reported [30]. Future iterations of apps such as the Vire app and ToDo-CR program may benefit from providing specific advice to reach such targets and help with creating more substantial levels of change [67].

The waist-to-hip ratio (0.03) and BMI (1.64 kg/m^2^) significantly reduced in the intervention group; however, they were unlikely to be clinically significant changes. The intervention also had a statistically significant 6-cm reduction in waist circumference, which could be considered clinically significant after accounting for measurement error (≥2 cm) [68]. These results combined may indicate an overall lower central adiposity and lower cardiovascular disease risk profile, in line with previous app-based studies in CHD participants [25].

A high percentage of the intervention group did not complete a single *Do* message (24/60, 40%), which was used as the marker of engagement. There may have been a misunderstanding of the need to tick off the *Dos* after viewing them or, alternatively, the intervention did not appeal to them. Previous studies on cardiovascular disease and smartphone apps have reported similarly low engagement and adherence levels [25,62,69-71] and that levels tend to decrease with time in the intervention [30]. Similar studies have also reported technical difficulties as a key reason for low engagement [30,62,69,70,72]. Low levels of engagement, as seen in this study, could result in an engagement level that is insufficient to achieve the intended effect [73], with increasing evidence that digital health apps for chronic disease self-management require ongoing patient engagement as a key determinant of overall clinical impact [74-77].

In line with the feasibility trial preceding this study [28], not having a smartphone was a major reason for exclusion. Among those who declined to participate, the main reason provided was not being interested in smartphone apps. Our study was consistent with previous studies, with people declining to participate being significantly older [28,30] and younger cardiac rehabilitation participants being more likely to use smartphones [22,23,30,78]. Patient preference for the type of intervention may be key to their success [26,67]. In a study comparing multiple delivery methods for cardiac rehabilitation including app-based cardiac rehabilitation, approximately 25% of the group declining the app-based approach listed technology issues as the main reason for nonparticipation [26]. Perhaps key messaging moving forward is that those who want to use smartphone apps will engage regardless of age [67].

Although smartphone apps have the potential to be a cost-effective solution [79], further work is required to ensure that the health benefits offset the initial increased costs of setting up and implementing such interventions. There is 1 relevant comparison study that evaluated the cost-effectiveness of a smartphone app in people with heart failure [80]. This number highlights the relative infancy of research in this area. The CardioManager app achieved greater savings in the management of heart failure with an ICER of €9000 (US $9595.8) per patient, equating to large reductions in costs associated with hospital admissions [80]. Future research in this area would benefit from reporting the economic evaluations of smartphone apps to determine their cost-effectiveness and improve research translation and real-world implementation.

### Strengths and Limitations

This study has several strengths being one of the first published studies to explore the impact of a sedentary behavior change smartphone app on hospital admissions and its cost-effectiveness. Device-measured sedentary behavior was analyzed, personalization and clinical guidelines were championed, and the study included both public and private hospitals. This is critical for improving the implementation of research apps in real-world settings.

Despite these strengths, there were several limitations. First, this trial was interrupted during the COVID-19 pandemic, impacting the recruitment and follow-up of participants and ultimately resulting in incomplete data. The number of participants who completed outcomes remotely have been reported, and sensitivity analyses were completed in line with guidelines for reporting trials affected by the pandemic [34]. No participants reported dropping out owing to the pandemic; however, there were difficulties recruiting, and hence, the target sample was not reached. Despite this limitation, the final dropout rate was lower than expected; therefore, the required sample size was maintained. Second, the majority of participants were male, tertiary educated, and working, and those excluded were significantly older, had more severe diagnoses, and were predominantly from the public health system, limiting generalizability. There were multiple assessors that may have affected the measurement error of outcomes such as waist circumference. Measuring engagement with the Vire app and ToDo-CR program was also limited. Further investigation into app engagement using back-end data is needed to better understand the relationship between app use and changes in sedentary behavior. Although half of the participants had experience using physical activity trackers, there is evidence that inexperienced users may not use all app features and therefore may not obtain the full anticipated benefits of behavior change smartphone apps [81]. In addition, although the use of ActiGraph accelerometers provides an objective measure of sedentary behavior, they are less sensitive to postural changes than devices such as ActiPal [13]. Future research would benefit from the use of inclinometers to detect changes from sitting to lying to standing and from combining this information with heart rate monitors and GPS technology to better inform the holistic picture of individual behavior surrounding sedentariness [82]. Finally, bringing the last value forward for missing data and the lack of participant blinding inherently introduced bias to the methods used.

### Conclusions

It does not appear that the Vire app and ToDo-CR program targeting sedentary behavior is an outcome-effective or cost-effective solution to reduce all-cause hospital admissions or ED presentations in cardiac rehabilitation participants. Although those using the Vire app and the ToDo-CR program had more hospital admissions, these admissions were less costly. Further research is warranted to improve engagement and implementation with age appearing to be less of an impacting factor and prior experience with apps correlating more with engagement. This type of intervention may work as a better resource for those not already attending cardiac rehabilitation, who are at greater risk of hospitalizations, to influence sedentary behavior and potentially reduce costs associated with cardiac-related hospital admissions.

## Appendix

Multimedia Appendix 1 Supplementary tables for the ToDo-CR randomized controlled trial.

Multimedia Appendix 2 CONSORT-eHEALTH checklist (V 1.6.1).

## Abbreviations

AR-DRG: Australian Refined Diagnosis Related Group
AQoL: Assessment of Quality of Life
CHD: coronary heart disease
CONSORT: Consolidated Standards of Reporting Trials
cpm: counts per minute
ED: emergency department
ICER: incremental cost-effectiveness ratio
VM: vector magnitude
