# Supplementary material for: Testing the Effect of a Smartphone App on Hospital Admissions and Sedentary Behavior in Cardiac Rehabilitation Participants: ToDo-CR Randomized Controlled Trial
Source: JMIR Mhealth Uhealth. 2023 Oct 3;11:e48229. doi: 10.2196/48229 (PMC10582808; doi:10.2196/48229)
Supplement: Multimedia Appendix 1 [file mhealth_v11i1e48229_app1.docx]

# Multimedia Appendix 1

### Table S1. Difference between those who agreed to participate versus those who declined

| **Characteristic** | **Agreed**  **(n=120)** | **Excluded/Declined**  **(n=114)** | ***P* value** |
| --- | --- | --- | --- |
| Age *(years)* | 62.61 (10.10) | 66.57 (10.38) | .003 |
| Sex *(male)*, n (%) | 93 (78) | 80 (70) | .26 |
| Diagnosis, n (%)  Stable coronary heart disease  CABG  PCI  Myocardial infarction  Myocardial infarction + PCI | 3 (3)  24 (20  59 (49)  3 (3)  31 (26) | 2 (2)  27 (24)  36 (32)  14 (12)  35 (31) | .01 |
| Hospital *(public)*, n (%) | 75 (63) | 94 (83) | .001 |

Independent samples t-test for continuous and chi-square for categorical

CABG, coronary artery bypass graft; PCI, percutaneous coronary intervention

### Table S2. Reason for exclusion or declining to participate based on age

| **Reason provided** | **Age (years)** | | | | |
| --- | --- | --- | --- | --- | --- |
|  | **40-49** | **50-59** | **60-69** | **≥70** | **Total, n (%)** |
| **Exclusion** |  |  |  |  |  |
| No smartphone | 0 | 0 | 4 | 10 | 14 (12) |
| Smartphone not compatible | 1 | 1 | 2 | 3 | 7 (6) |
| **Decline** |  |  |  |  |  |
| Not interested in smartphones or apps | 1 | 3 | 7 | 17 | 28 (25) |
| Not interested in participating in research | 3 | 3 | 5 | 6 | 17 (15) |
| Declined with no reason provided | 1 | 0 | 7 | 8 | 16 (14) |
| Too busy with work commitments | 0 | 5 | 3 | 2 | 10 (9) |
| Unable to use smartwatch or apps due to work privacy | 2 | 1 | 2 | 0 | 5 (4) |
| Felt it was too complicated for them | 0 | 0 | 1 | 4 | 5 (4) |
| Too overwhelmed with managing own health | 0 | 0 | 1 | 3 | 4 (4) |
| Participating in another study | 0 | 1 | 1 | 0 | 2 (2) |
| Satisfied with using commercially available smartwatch and/or apps | 0 | 1 | 0 | 1 | 2 (2) |
| Busy with carers commitments | 0 | 0 | 1 | 1 | 2 (2) |
| Already physically active and felt would not benefit from participating | 1 | 1 | 0 | 0 | 2 (2) |
| **Total** | 9 | 16 | 34 | 55 | 114 |

### Table S3. Comparison of baseline characteristics of those who dropped out and continued at 6- and 12-months

|  | **6-months** | | |  | **12-months** | | |
| --- | --- | --- | --- | --- | --- | --- | --- |
| **Characteristics** | **Continued**  **(n=113)** | **Dropouts**  **(n=7)** | ***P* value** |  | **Continued**  **(n=108)** | **Dropouts**  **(n=12)** | ***P* value** |
| Age *(years)*, mean (SD) | 62.34 (10.13) | 67.00 (9.11) | .24 |  | 62.39 (10.13) | 64.58 (10.02) | .48 |
| Sex *(male)*, n (%) | 87 (77) | 6 (86) | .59 |  | 83 (77) | 10 (83) | .61 |
| Education, n (%)  Primary  Secondary  Tertiary | 0  22 (20)  91 (80) | 0  3 (43)  4 (57) | .14 |  | 0  22 (20)  86 (80) | 0  3 (25)  9 (75) | .71 |
| Employment, n (%)  Full-time  Part-time  Unemployed  Voluntary work  Not in the labor force | 46 (41)  14 (12)  1 (1)  5 (4)  47 (42) | 2 (29)  0  0  0  5 (71) | .59 |  | 43 (40)  13 (12)  1 (1)  5 (5)  46 (43) | 5 (42)  1 (8)  0  0  6 (50) | .92 |
| Relationship status *(partner)*, n (%) | 96 (85) | 7 (100) | .27 |  | 93 (86) | 10 (83) | .79 |
| Previous experience with physical activity trackers, n (%)  Smartphone app  Smartwatch  Both  Neither | 20 (18)  23 (20)  11 (10)  59 (52) | 1 (14)  0  0  6 (86) | .32 |  | 19 (18)  22 (20)  11 (10)  56 (52) | 2 (17)  1 (8)  0  9 (75) | .38 |
| Diagnosis, n (%)  Stable coronary heart disease  CABG  PCI  Myocardial infarction  Myocardial infarction + PCI | 2 (2)  23 (20)  56 (50)  2 (2)  30 (27) | 1 (14)  1 (14)  3 (43)  1 (14)  1 (14) | .06 |  | 2 (2)  23 (21)  52 (48)  2 (2)  29 (27) | 1 (8)  1 (8)  7 (58)  1 (8)  2 (17) | .27 |
| BMI (kg/m^2^), mean (SD) | 28.72 (4.62) | 26.96 (5.04) | .33 |  | 28.76 (4.67) | 27.31 (4.33) | .30 |
| Waist circumference *(cm)*, mean (SD) | 103.71 (14.46) | 102.40 (17.20) | .84 |  | 103.62 (14.59) | 103.93 (14.41) | .95 |
| Waist-to-hip ratio, mean (SD) | 0.97 (0.10) | 0.95 (0.09) | .70 |  | 0.97 (0.09) | 1.00 (0.11) | .37 |
| Systolic blood pressure *(mmHg)*, mean (SD) | 127.44 (13.94) | 128.43 (14.47) | .86 |  | 127.45 (14.06) | 127.92 (13.06) | .91 |
| Diastolic blood pressure *(mmHg)*, mean (SD) | 74.49 (8.26) | 77.29 (4.9) | .38 |  | 74.64 (8.20) | 74.75 (7.81) | .97 |
| 6-minute walk test distance *(m)*, mean (SD) | 512.80 (86.66) | 540.20 (74.05) | .49 |  | 512.28 (84.37) | 531.40 (103.76) | .51 |
| ActiGraph, mean (SD)  SB *(mins per day)*  Percentage of SB per day *(SB per wear time)*  Average duration of SB bouts *(mins)*  Number of SB bouts per day  Number of SB breaks per day  MVPA *(mins per day)*  LPA *(mins per day)*  VM *(counts per day)*  Steps *(per day)*  Wear time *(mins per day)* | 591.94 (147.92)  67.56% (9.47)  21.35 (3.48)  13.39 (5.69)  12.49 (5.69)  50.51 (31.32)  231.19 (68.77)  413775.65 (175331.42)  6366.48 (3234.05)  873.62 (141.74) | 526.35 (85.51)  66.55% (12.52)  20.50 (3.46)  12.63 (4.72)  11.64 (4.72)  40.69 (16.68)  227.27 (94.92)  368439.47 (141600.51)  5250.22 (2171.87)  794.40 (34.73) | .33  .84  .59  .74  .74  .49  .90  .57  .45  .22 |  | 590.95 (150.28)  67.33% (9.55)  21.29 (3.36)  13.48 (5.77)  12.48 (5.77)  51.44 (31.43)  231.08 (68.88)  417941.27 (176104.23)  6440.32 (3255.70)  873.46 (143.34) | 566.75 (87.70)  68.39% (10.22)  21.59 (4.74)  13.16 (3.98)  12.16 (3.98)  34.53 (17.45)  230.23 (81.72)  341378.60 (130896.69)  4909.57 (2033.75)  831.44 (82.62) | .64  .75  .81  .87  .87  .12  .97  .21  .17  .39 |
| AQoL-6D  Utility Instrument, mean (SD) | 81.27 (9.7) | 76.31 (14.3) | .21 |  | 81.59 (8.96) | 75.53 (16.37) | .23 |
| HADS  Anxiety, mean (SD)  Depression, mean (SD) | 4.74 (3.09)  3.01 (2.64) | 5.57 (4.54)  5.00 (4.04) | .50  .06 |  | 4.68 (3.04)  3.01 (2.60) | 5.75 (4.22)  4.17 (3.86) | .27  .16 |
| URICA-E2  Readiness to change, mean (SD) | 5.13 (1.11) | 4.95 (1.13) | .69 |  | 5.09 (1.12) | 5.32 (1.00) | .50 |

*P* values reported from independent samples T-test and Pearson Chi Square as appropriate.

SB, sedentary behavior; BMI, body mass index; MVPA, moderate-to-vigorous physical activity; LPA, light-intensity physical activity; VM, vector magnitude; HADS, Hospital Anxiety and Depression Scale.

### Table S4. Survival analysis of time to first admission and emergency department presentation using Cox regression model

| **Dependent variable^a^** | **Model 1**^b^ | | |  | **Model 2**^c^ | | |  | **Model 3**^d^ | | |  |
| --- | --- | --- | --- | --- | --- | --- | --- | --- | --- | --- | --- | --- |
|  | **HR**^e^ | **95% CI** | ***P* value** |  | **HR** | **95% CI** | ***P* value** |  | **HR** | **95% CI** | ***P* value** |  |
| Non-elective all-cause hospital admissions | 1.24 | 0.54 to 2.87 | .62 |  | 1.27 | 0.54 to 2.98 | .58 |  | 1.52 | 0.63 to 3.67 | .35 |  |
| Non-elective cardiac-related hospital admissions^f^ | 2.13 | 0.64 to 7.09 | .22 |  | 2.21 | 0.66 to 7.46 | .20 |  | 3.14 | 0.90 to 10.99 | .07 |  |
| Emergency department presentations | 1.59 | 0.83 to 3.07 | .17 |  | 1.61 | 0.83 to 3.12 | .16 |  | 1.84 | 0.93 to 3.63 | .08 |  |

^a^Reference = control.

^b^Model 1: nil adjustments.

^c^Model 2: adjusted for age and sex.

^d^Model 3: adjusted for age, sex, diabetes, and presence of other chronic disease.

^e^HR, hazards ratio.

^f^Cardiac-related admission determined by Australian Refined Diagnosis Related Group codes (F01A to F10B, F12A to F12B, F14A to F19B, F22Z to F60B, F66A to F67B, and F69A to F76B).

### Table S5. Comparison of baseline, 6-month and 12-month sedentary behavior and physical activity measures

|  | Groups | | | | | | | |  | **Difference within groups** | | | | |  | **Difference between groups** | | |
| --- | --- | --- | --- | --- | --- | --- | --- | --- | --- | --- | --- | --- | --- | --- | --- | --- | --- | --- |
|  | Baseline | |  | **6-months** | |  | **12-months** | |  | **6-months minus Baseline** | |  | **12-months minus Baseline** | |  | **6-months** |  | **12-months** |
| **ActiGraph outcomes** | **Int (n=54)** | **Con (n=57)** |  | **Int (n=58)** | **Con (n=59)** |  | **Int (n=58)** | **Con (n=60)** |  | **Int** | **Con** |  | **Int** | **Con** |  | **Int - Con** |  | **Int - Con** |
| SB *(mins per day)* | 578.06 (118.26) | 599.34 (168.68) |  | 591.46 (151.43) | 608.61 (161.85) |  | 591.11 (152.91) | 623.91 (156.13) |  | 18.71 (115.84) | 15.19 (117.31) |  | 18.23 (116.87) | 29.19 (160.66) |  | -17.16 (-74.58 to 40.27) |  | -32.80 (-89.17 to 23.57) |
| Percentage of SB per day *(SB per wear time)* | 66.51% (9.67) | 68.28% (9.46) |  | 66.16% (10.19) | 67.29% (9.40) |  | 66.94% (10.64) | 67.87% (10.25) |  | -0.58 (7.71) | -0.57 (6.05) |  | 0.20 (9.48) | -0.28 (7.80) |  | -1.13 (-4.72 to 2.46) |  | -0.93 (-4.74 to 2.87) |
| Average duration of SB bouts *(mins)* | 21.31 (3.31) | 21.32 (3.63) |  | 21.60 (3.87) | 20.62 (3.22) |  | 21.62 (4.29) | 21.89 (4.43) |  | 0.40 (3.04) | -0.64 (3.00) |  | 0.49 (3.36) | 0.53 (2.56) |  | 0.98 (-0.32 to 2.28) |  | -0.27 (-1.87 to 1.32) |
| Number of SB bouts per day | 12.98 (4.65) | 13.90 (6.44) |  | 13.12 (5.71) | 13.96 (6.05) |  | 13.15 (5.86) | 13.99 (5.95) |  | 0.42 (4.23) | 0.36 (4.46) |  | 0.41 (4.44) | 0.28 (6.53) |  | -0.85 (-3.00 to 1.31) |  | -0.83 (-2.99 to 1.32) |
| Number of SB breaks per day | 11.98 (4.65) | 12.90 (6.44) |  | 12.12 (5.70) | 12.97 (6.04) |  | 12.16 (5.84) | 12.99 (5.95) |  | 0.42 (4.23) | 0.36 (4.46) |  | 0.42 (4.44) | 0.28 (6.53) |  | -0.85 (-3.00 to 1.31) |  | -0.82 (-2.97 to 1.33) |
| MVPA *(mins per day)* | 51.45 (32.65) | 48.76 (29.23) |  | 50.72 (34.39) | 54.38 (29.39) |  | 48.43 (33.47) | 51.76 (31.17) |  | 0.41 (25.53) | 5.64 (21.11) |  | -1.77 (31.20) | 3.31 (20.98) |  | -3.65 (-15.36 to 8.05) |  | -3.33 (-15.12 to 8.46) |
| LPA *(mins per day)* | 238.99 (72.11) | 223.45 (66.87) |  | 248.21 (76.20) | 236.63 (74.65) |  | 239.90 (74.29) | 238.39 (79.61) |  | 13.11 (64.14) | 11.42 (48.73) |  | 4.40 (70.55) | 15.26 (53.69)^a^ |  | 11.57 (-16.05 to 39.20) |  | 1.51 (-26.59 to 29.61) |
| VM *(counts per day)* | 428828.18 (189697.79) | 395538.51 (156973.87) |  | 430249.31 (186984.11) | 428994.54 (161503.86) |  | 412993.95 (187866.50) | 420387.01 (170242.21) |  | 9386.36 (149751.66) | 32550.66 (105770.84)^b^ |  | -8148.63 (183095.83) | 27310.62 (114888.68) |  | 1254.78 (-62694.55 to 65204.10) |  | -7393.05 (-72720.57 to 57934.46) |
| Steps *(per day)* | 6592.52 (3471.72) | 6054.42 (2916.07) |  | 6484.16 (3346.86) | 6351.24 (2663.40) |  | 6072.18 (2981.38) | 6323.91 (2920.74) |  | -0.52 (2658.82) | 277.59 (2239.20) |  | -421.83 (2788.25) | 247.30 (1857.03) |  | 132.92 (-973.76 to 1239.60) |  | -251.73 (-1327.90 to 824.43) |
| Wear time *(mins per day)* | 868.48 (120.00) | 871.54 (157.04) |  | 890.43 (160.20) | 899.66 (164.97) |  | 879.47 (151.20) | 914.82 (142.63) |  | 32.30 (136.37) | 32.30 (131.48)^b^ |  | 20.89 (120.22) | 48.56 (148.33)^c^ |  | -9.23 (-68.79 to 50.33) |  | -35.35 (-88.93 to 18.23)^d^ |
| Accelerometer worn during COVID-19 lockdowns, n (%) | 4 (7) | 3 (5) |  | 11 (19) | 12 (20) |  | 1 (2) | 1 (2) |  |  |  |  |  |  |  |  |  |  |

Mean (SD) of groups, mean (SD) difference within groups, and mean (95% CI) difference between groups.

Intention to treat was used.

^a^Paired samples t-test within groups, *P* < .05

^b^Wilcoxon test within groups, *P* < .05

^c^Wilcoxon test within groups, *P* < .001

^d^Mann-Whitney U test between groups, *P* < .05

Int, Intervention group; Con, control group; SB, sedentary behavior; MVPA, moderate-to-vigorous physical activity; LPA, light-intensity physical activity; VM, vector magnitude.

### Table S6. Comparison of those in the intervention group who already had a physical activity tracker (smartphone app or smartwatch) versus those without prior to the trial

|  | Baseline | |  | **6-months** | |  | **12-months** |  |  |
| --- | --- | --- | --- | --- | --- | --- | --- | --- | --- |
| **Outcomes** | **No tracker – Tracker**  **(n=30) (n=30)** | ***P* value** |  | **No tracker - Tracker** | ***P* value** |  | **No tracker - Tracker** | ***P* value** |  |
| ActiGraph |  |  |  |  |  |  |  |  |  |
| SB *(mins per day)* | 8.73 (-56.47 to 73.93) | .79 |  | 65.22 (-13.29 to 143.72) | .10 |  | 72.93 (-5.90 to 151.75) | .07 |  |
| Percentage of SB per day *(SB per wear time)* | 4.33 (-0.87 to 9.53) | .10 |  | 6.13 (0.97 to 11.28) | .02 |  | 4.33 (-1.19 to 9.86) | .12 |  |
| Average duration of SB bouts *(mins)* | 1.92 (0.18 to 3.67) | .03 |  | 2.32 (0.36 to 4.27) | .02 |  | 2.22 (0.02 to 4.42) | .05 |  |
| Number of SB bouts per day | 0.56 (-2.01 to 3.12) | .66 |  | 2.30 (-0.66 to 5.27) | .13 |  | 2.33 (-0.71 to 5.38) | .13 |  |
| Number of SB breaks per day | 0.56 (-2.01 to 3.12) | .66 |  | 2.30 (-0.66 to 5.27) | .13 |  | 2.33 (-0.71 to 5.36) | .13 |  |
| MVPA *(mins per day)* | -20.44 (-37.54 to -3.35) | .02 |  | -18.11 (-35.72 to -0.50) | .04 |  | -9.87 (-27.45 to 7.70) | .27 |  |
| LPA *(mins per day)* | -32.34 (-71.09 to 6.42) | .10 |  | -26.80 (-66.63 to 13.02) | .18 |  | -13.61 (-52.89 to 25.68) | .49 |  |
| VM *(counts per day)* | -125,740.79 (-223,069.30 to -28,412.27) | .01 |  | -109,538.04 (-203,492.74 to -15,583.34) | .02 |  | -57,082.79 (-155,675.72 to 41,510.14) | .25 |  |
| Steps *(per day)* | -2135.55 (-3956.54 to -314.56) | .02 |  | -2012.05 (-3705.89 to -318.21) | .02 |  | -1249.07 (-2796.68 to 298.53) | .11 |  |

Mean (95% CI) difference between groups

SB, sedentary behavior; MVPA, moderate-to-vigorous physical activity; LPA, light-intensity physical activity; VM, vector magnitude.

### Table S7. Comparison of those in the control group with a physical activity tracker (smartphone app or smartwatch) versus those without

|  | Baseline | |  | **6-months** | |  | **12-months** |  |  |
| --- | --- | --- | --- | --- | --- | --- | --- | --- | --- |
| **Outcomes** | **No tracker – Tracker**  **(n=35) (n=25)** | ***P* value** |  | **No tracker - Tracker** | ***P* value** |  | **No tracker - Tracker** | ***P* value** |  |
| ActiGraph |  |  |  |  |  |  |  |  |  |
| SB *(mins per day)* | 20.32 (-71.03 to 111.66) | .66 |  | -7.58 (-94.20 to 79.04) | .86 |  | -32.20 (-114.30 to 49.91) | .44 |  |
| Percentage of SB per day *(SB per wear time)* | 2.43 (-2.66 to 7.52) | .34 |  | 0.86 (-4.17 to 5.89) | .73 |  | -0.42 (-5.83 to 5.00) | .88 |  |
| Average duration of SB bouts *(mins)* | 0.94 (-0.85 to 2.72) | .30 |  | 0.52 (-1.19 to 2.24) | .54 |  | 2.20 (-0.07 to 4.47) | .06 |  |
| Number of SB bouts per day | 1.34 (-2.14 to 4.82) | .44 |  | 0.68 (-2.55 to 3.92) | .68 |  | -0.73 (-3.87 to 2.41) | .65 |  |
| Number of SB breaks per day | 1.34 (-2.14 to 4.82) | .44 |  | 0.68 (-2.55 to 3.92) | .68 |  | -0.73 (-3.87 to 2.41) | .65 |  |
| MVPA *(mins per day)* | -9.09 (-24.76 to 6.57) | .25 |  | -4.24 (-19.93 to 11.45) | .59 |  | 2.10 (-14.37 to 18.57) | .80 |  |
| LPA *(mins per day)* | -14.38 (-50.45 to 21.69) | .43 |  | -8.94 (-46.57 to 28.69) | .64 |  | -4.02 (-43.64 to 35.60) | .84 |  |
| VM *(counts per day)* | -48,999.15 (-133,120.86 to 35,122.55) | .25 |  | -18,572.42 (-104,891.11 to 67,746.27) | .67 |  | 16,888.87 (-73,003.90 to 106,781.64) | .71 |  |
| Steps *(per day)* | -1065.88 (-2777.50 to 645.75) | .21 |  | -218.89 (-1643.52 to 1205.75) | .76 |  | 221.43 (-1321.59 to 1764.45) | .78 |  |

Mean (95% CI) difference between groups

SB, sedentary behavior; MVPA, moderate-to-vigorous physical activity; LPA, light-intensity physical activity; VM, vector magnitude.

### Table S8. Comparison of baseline, 6- and 12-month secondary measures

|  | Groups | | | | | | | |  | **Difference within groups** | | | | |  | **Difference between groups** | | |
| --- | --- | --- | --- | --- | --- | --- | --- | --- | --- | --- | --- | --- | --- | --- | --- | --- | --- | --- |
|  | Baseline | |  | **6-months** | |  | **12-months** | |  | **6-months minus Baseline** | |  | **12-months minus Baseline** | |  | **6-months** |  | **12-months** |
| **Outcome** | **Int (n=60)** | **Con (n=60)** |  | **Int (n=60)** | **Con (n=60)** |  | **Int (n=60)** | **Con (n=60)** |  | **Int** | **Con** |  | **Int** | **Con** |  | **Int - Con** |  | **Int - Con** |
| BMI *(kg/m2)* | 27.94 (4.80) | 29.30 (4.40) |  | 27.74 (5.08) | 29.32 (4.40) |  | 28.33 (4.87) | 29.70 (4.37) |  | -0.20 (1.29) | 0.03 (1.35) |  | 0.39 (1.35)^c^ | 0.40 (1.59)^c^ |  | -1.59 (-3.31 to 0.13)^f^ |  | -1.37 (-3.04 to 0.30) |
| Waist circumference *(cm)* | 100.51 (15.36) | 106.85 (12.96) |  | 98.97 (15.15) | 104.60 (14.06) |  | 100.86 (14.24) | 106.64 (12.57) |  | -1.43 (4.73)^a^ | -2.47 (6.59)^a^ |  | 0.56 (6.16) | -0.22 (6.20) |  | -5.63 (-10.98 to -0.28)^e^ |  | -5.78 (-10.67 to -0.89)^f^ |
| Waist-to-hip ratio | 0.95 (0.11) | 0.99 (0.08) |  | 0.94 (0.10) | 0.97 (0.09) |  | 0.95 (0.09) | 0.98 (0.09) |  | -0.01 (0.05) | -0.02 (0.06)^c^ |  | 0.00 (0.05) | -0.01 (0.06) |  | -0.03 (-0.07 to 0.01) |  | -0.03 (-0.06 to 0.00) |
| Systolic blood pressure *(mmHg)* | 126.65 (13.30) | 128.36 (14.57) |  | 130.88 (13.47) | 131.18 (16.57) |  | 130.28 (14.26) | 132.45 (14.12) |  | 4.23 (11.49)^a^ | 3.02 (14.06) |  | 3.63 (11.98)^c^ | 4.39 (14.11)^a^ |  | -0.30 (-5.76 to 5.16) |  | -2.17 (-7.30 to 2.96) |
| Diastolic blood pressure *(mmHg)* | 76.00 (8.19) | 73.29 (7.89) |  | 78.63 (7.86) | 76.42 (9.11) |  | 78.30 (7.73) | 77. 65 (8.66) |  | 2.63 (7.43)^c^ | 3.15 (8.59)^c^ |  | 2.30 (8.54)^a^ | 4.41 (7.75)^b^ |  | 2.22 (-0.86 to 5.29) |  | 0.65 (-2.32 to 3.62) |
| 6-minute walk test distance *(m)* | 514.67 (95.85) | 513.48 (75.34) |  | 547.33 (102.66) | 549.43 (84.06) |  | 564.95 (87.22) | 570.22 (89.98) |  | 33.00 (55.00)^b^ | 40.75 (60.78)^d^ |  | 52.12 (64.33)^b^ | 62.35 (71.51)^b^ |  | -2.10 (-36.38 to 32.18) |  | -5.27 (-37.60 to 27.06) |
| AQoL-6D (*0-100*) |  |  |  |  |  |  |  |  |  |  |  |  |  |  |  |  |  |  |
| Utility instrument | 79.26 (9.99) | 82.70 (9.82) |  | 82.13 (9.50) | 85.15 (10.00) |  | 83.65 (10.63) | 85.17 (11.54) |  | 2.87 (6.60)^c^ | 2.45 (5.52)^c^ |  | 4.39 (7.45)^d^ | 2.47 (8.58)^d^ |  | -3.02 (-6.55 to 0.51)^f^ |  | -1.52 (-5.53 to 2.49) |
| Independent living | 85.56 (14.72) | 89.07 (12.40) |  | 90.56 (10.89) | 93.61 (10.06) |  | 92.41 (11.20) | 93.15 (12.16) |  | 5.00 (14.16)^c^ | 4.54 (10.94)^c^ |  | 6.85 (12.75)^d^ | 4.07 (15.27)^c^ |  | -3.05 (-6.85 to 0.74)^f^ |  | -0.74 (-4.97 to 3.49) |
| Relationships | 86.50 (14.59) | 90.33 (12.48) |  | 90.67 (12.47) | 92.83 (11.36) |  | 91.00 (12.45) | 91.17 (13.91) |  | 4.17 (11.83)^c^ | 2.50 (7.04)^c^ |  | 4.50 (11.85)^c^ | 0.83 (12.25) |  | -2.17 (-6.48 to 2.15) |  | -0.17 (-4.94 to 4.61) |
| Mental health | 73.85 (15.11) | 78.33 (14.39) |  | 74.58 (15.98) | 80.21 (12.30) |  | 77.71 (16.53) | 81.04 (13.52) |  | 0.73 (9.90) | 1.88 (10.05) |  | 3.85 (12.29)^c^ | 2.71 (12.57) |  | -5.63 (-10.78 to -0.47)^b^ |  | -3.33 (-8.79 to 2.13) |
| Coping | 70.28 (11.62) | 76.11 (15.07) |  | 74.17 (11.76) | 77.64 (14.02) |  | 76.25 (14.21) | 79.31 (15.07) |  | 3.89 (9.39)^c^ | 1.53 (8.33) |  | 5.97 (12.75)^d^ | 3.19 (10.42)^c^ |  | -3.47 (-8.15 to 1.21) |  | -3.06 (-8.35 to 2.24) |
| Pain | 75.00 (20.38) | 80.67 (16.76) |  | 78.17 (18.27) | 83.00 (17.50) |  | 78.33 (20.84) | 80.67 (20.90) |  | 3.17 (12.42) | 2.33 (15.88) |  | 3.33 (17.04) | 0.00 (17.47) |  | -4.83 (-11.30 to 1.63) |  | -2.33 (-9.88 to 5.21) |
| Senses | 83.21 (10.26) | 81.03 (10.74) |  | 83.59 (10.55) | 82.18 (11.20) |  | 84.10 (11.67) | 83.46 (11.84) |  | 0.38 (7.42) | 1.15 (8.59) |  | 0.90 (7.90) | 2.44 (8.56)^c^ |  | 1.41 (-2.52 to 5.34) |  | 0.64 (-3.61 to 4.89) |
| HADS (*0-21*) |  |  |  |  |  |  |  |  |  |  |  |  |  |  |  |  |  |  |
| Anxiety | 5.39 (3.23) | 4.20 (3.03) |  | 4.73 (3.24) | 3.85 (2.91) |  | 4.63 (3.25) | 3.70 (2.76) |  | -0.64 (2.06)^c^ | -0.35 (2.07) |  | -0.73 (1.84)^a^ | -0.50 (2.05) |  | 0.88 (-0.23 to 2.00) |  | 0.93 (-0.16 to 2.02) |
| Depression | 3.14 (2.58) | 3.12 (2.95) |  | 2.63 (2.64) | 2.53 (2.87) |  | 2.75 (2.69) | 2.18 (2.63) |  | -0.46 (1.69)^c^ | -0.58 (1.92)^c^ |  | -0.36 (1.82) | -0.93 (2.06)^c^ |  | 0.10 (-0.90 to 1.10) |  | 0.57 (-0.40 to 1.53) |
| URICA-E2 (*0-14*) |  |  |  |  |  |  |  |  |  |  |  |  |  |  |  |  |  |  |
| Readiness to change | 5.14 (1.08) | 5.09 (1.13) |  | 5.45 (1.07) | 5.19 (1.28) |  | 5.20 (0.96) | 5.25 (1.79) |  | 0.27 (1.09) | 0.10 (1.14) |  | 0.05 (1.18) | 0.15 (1.63) |  | 0.26 (-0.17 to 0.69) |  | -0.05 (-0.57 to 0.47) |
| Assessment completed using telehealth, n (%) | 5 (8) | 7 (12) |  | 16 (27) | 15 (25) |  | 10 (17) | 11 (18) |  |  |  |  |  |  |  |  |  |  |

Mean (SD) of groups, mean (SD) difference within groups, and mean (95% CI) difference between groups.

Intention to treat was used.

^a^Paired samples t-test within groups, *P* < .05

^b^Paired samples t-test within groups, *P* < .001

^c^Wilcoxon test within groups, *P* < .05

^d^Wilcoxon test within groups, *P* < .001

^e^Independent samples t-test between groups, *P* < .05

^f^Mann-Whitney U test between groups, *P* < .05

Int, Intervention group; Con, control group; SB, sedentary behavior; BMI, body mass index; HADS, Hospital Anxiety and Depression Scale.

### Table S9. Difference between secondary outcomes over 6- and 12-months between groups using linear mixed effects models

|  | **Model 1**^b^ | | | | | | | | |  | **Model 2**^c^ | | | | | | | | |  | **Model 3**^d^ | | | | | | | | |
| --- | --- | --- | --- | --- | --- | --- | --- | --- | --- | --- | --- | --- | --- | --- | --- | --- | --- | --- | --- | --- | --- | --- | --- | --- | --- | --- | --- | --- | --- |
|  | **6-months** | | | |  | **12-months** | | | |  | **6-months** | | | |  | **12-months** | | | |  | **6-months** | | | |  | **12-months** | | | |
| **Dependent variable^a^** | **β** | **95% CI** | ***P* value** | **ICC** |  | **β** | **95% CI** | ***P* value** | **ICC** |  | **β** | **95% CI** | ***P* value** | **ICC** |  | **β** | **95% CI** | ***P* value** | **ICC** |  | **β** | **95% CI** | ***P* value** | **ICC** |  | **β** | **95% CI** | ***P* value** | **ICC** |
| BMI *(kg/m2)* | 1.47 | -0.19 to 0.33 | .08 | 0.96 |  | 1.44 | -0.21 to 3.09 | .09 | 0.96 |  | 1.43 | -0.22 to 3.08 | .09 | 0.96 |  | 1.41 | -0.23 to 3.05 | .09 | 0.96 |  | 1.64 | 0.003 to 3.27 | .05 | 0.96 |  | 1.62 | -0.008 to 3.24 | .05 | 0.96 |
| Waist circumference *(cm)* | 6.09 | 1.01 to 11.18 | .01 | 0.92 |  | 6.02 | 1.10 to 10.94 | .02 | 0.92 |  | 5.19 | 0.50 to 9.87 | .03 | 0.90 |  | 5.21 | 0.67 to 9.74 | .03 | 0.91 |  | 5.90 | 1.29 to 10.50 | .01 | 0.90 |  | 5.81 | 1.32 to 10.30 | .01 | 0.91 |
| Waist-to-hip ratio | 0.04 | 0.001 to 0.07 | .04 | 0.84 |  | 0.03 | 0.001 to 0.07 | .04 | 0.85 |  | 0.03 | -0.002 to 0.06 | .06 | 0.77 |  | 0.03 | -0.001 to 0.06 | .06 | 0.80 |  | 0.03 | 0.004 to 0.06 | .03 | 0.76 |  | 0.03 | 0.003 to 0.06 | .03 | 0.79 |
| Systolic blood pressure *(mmHg)* | 0.94 | -3.74 to 5.62 | .69 | 0.61 |  | 1.33 | -3.16 to 5.81 | .56 | 0.63 |  | -0.83 | -5.10 to 3.44 | .70 | 0.54 |  | -0.29 | -4.42 to 3.83 | .89 | 0.57 |  | -0.87 | -5.16 to 3.41 | .69 | 0.54 |  | -0.24 | -4.39 to 3.91 | .91 | 0.57 |
| Diastolic blood pressure *(mmHg)* | -2.89 | -4.33 to 0.13 | .001 | 0.53 |  | -1.86 | -4.36 to 0.63 | .14 | 0.56 |  | -2.45 | -4.97 to 0.07 | .06 | 0.50 |  | -1.71 | -4.11 to 0.69 | .16 | 0.53 |  | -2.67 | -5.19 to -0.15 | .04 | 0.49 |  | -1.89 | -4.30 to 0.52 | .12 | 0.52 |
| 6-minute walk test distance *(m)* | -1.03 | -32.15 to -25.28 | .95 | 0.79 |  | 1.32 | -28.86 to 31.50 | .93 | 0.80 |  | 1.55 | -27.82 to 30.91 | .92 | 0.77 |  | 5.10 | -23.23 to 33.43 | .72 | 0.77 |  | 2.91 | -26.49 to 32.31 | .85 | 0.76 |  | 6.07 | -22.30 to 34.43 | .67 | 0.76 |
| AQoL-6D (*0-100*) |  |  |  |  |  |  |  |  |  |  |  |  |  |  |  |  |  |  |  |  |  |  |  |  |  |  |  |  |  |
| Utility instrument | 3.23 | -0.12 to 6.58 | .06 | 0.81 |  | 2.66 | -0.73 to 6.05 | .12 | 0.77 |  | 3.89 | 0.57 to 7.21 | .02 | 0.80 |  | 3.50 | -2.02 to 6.80 | .04 | 0.75 |  | 3.70 | 0.45 to 6.95 | .03 | 0.79 |  | 3.30 | 0.03 to 6.58 | .05 | 0.74 |
| Independent living | 3.29 | -0.43 to 7.00 | .08 | 0.46 |  | 2.38 | -1.12 to 5.87 | .18 | 0.49 |  | 4.01 | 0.33 to 7.69 | .03 | 0.44 |  | 3.29 | -0.10 to 6.68 | .06 | 0.46 |  | 3.88 | 0.30 to 7.46 | .03 | 0.41 |  | 3.04 | -0.28 to 6.37 | .07 | 0.44 |
| Relationships | 3.00 | -1.24 to 7.24 | .16 | 0.71 |  | 2.11 | -1.97 to 6.19 | .31 | 0.66 |  | 3.54 | -0.71 to 7.78 | .10 | 0.70 |  | 2.85 | -1.20 to 6.90 | .17 | 0.65 |  | 3.39 | -0.86 to 7.64 | .12 | 0.70 |  | 2.80 | -1.26 to 6.86 | .17 | 0.65 |
| Mental health | 5.05 | 0.17 to 9.94 | .04 | 0.76 |  | 4.48 | -0.26 to 9.22 | .06 | 0.71 |  | 5.06 | 0.11 to 10.01 | .05 | 0.76 |  | 4.78 | -0.02 to 9.57 | .05 | 0.71 |  | 5.10 | 0.13 to 10.07 | .04 | 0.76 |  | 4.88 | 0.07 to 9.70 | .05 | 0.71 |
| Coping | 4.65 | 0.20 to 9.11 | .04 | 0.77 |  | 4.12 | -0.31 to 8.55 | .07 | 0.72 |  | 5.14 | 0.66 to 9.62 | .03 | 0.77 |  | 4.75 | 0.31 to 9.18 | .04 | 0.71 |  | 4.96 | 0.46 to 9.46 | .03 | 0.77 |  | 4.46 | 0.01 to 8.92 | .05 | 0.71 |
| Pain | 5.25 | -0.78 to 11.28 | .09 | 0.70 |  | 4.28 | -1.82 to 10.38 | .17 | 0.68 |  | 6.27 | 0.27 to 12.27 | .04 | 0.69 |  | 5.54 | -0.46 to 11.55 | .07 | 0.67 |  | 6.18 | 0.49 to 11.88 | .03 | 0.65 |  | 5.46 | -0.35 to 11.27 | .07 | 0.64 |
| Senses | -1.80 | -5.35 to 1.76 | .32 | 0.72 |  | -1.37 | -4.98 to 2.25 | .46 | 0.74 |  | -0.45 | -3.71 to 2.82 | .79 | 0.67 |  | -0.02 | -3.36 to 3.32 | .99 | 0.70 |  | -1.10 | -4.25 to 2.05 | .49 | 0.64 |  | -0.62 | -3.88 to 2.64 | .71 | 0.68 |
| HADS (*0-21*) |  |  |  |  |  |  |  |  |  |  |  |  |  |  |  |  |  |  |  |  |  |  |  |  |  |  |  |  |  |
| Anxiety | -1.03 | -2.07 to 0.02 | .06 | 0.78 |  | -1.01 | -2.04 to 0.01 | .05 | 0.80 |  | -1.02 | -2.08 to 0.04 | .06 | 0.78 |  | -0.98 | -2.02 to 0.06 | .06 | 0.80 |  | -0.98 | -2.05 to 0.09 | .07 | 0.78 |  | -0.94 | -1.99 to 0.11 | .08 | 0.80 |
| Depression | -0.03 | -0.97 to 0.90 | .94 | 0.79 |  | -0.23 | -1.14 to 0.68 | .62 | 0.78 |  | -0.19 | -1.11 to 0.74 | .69 | 0.78 |  | -0.40 | -1.29 to 0.50 | .38 | 0.77 |  | -0.17 | -1.11 to 0.76 | .71 | 0.78 |  | -0.37 | -1.27 to 0.53 | .42 | 0.77 |
| URICA-E2 (*0-14*) |  |  |  |  |  |  |  |  |  |  |  |  |  |  |  |  |  |  |  |  |  |  |  |  |  |  |  |  |  |
| Readiness to change | -0.17 | -0.53 to 0.19 | .34 | 0.54 |  | -0.10 | -0.46 to 0.27 | .60 | 0.48 |  | -0.19 | -0.55 to 0.17 | .30 | 0.53 |  | -0.12 | -0.48 to 0.25 | .53 | 0.47 |  | -0.14 | -0.50 to 0.22 | .44 | 0.51 |  | -0.08 | -0.44 to 0.28 | .66 | 0.46 |

^a^ Reference = Intervention.

^b^Model 1: nil adjustments.

^c^Model 2: adjusted for age and sex.

^d^Model 3: adjusted for age, sex, employment, and education.

BMI, body mass index; ICC, intraclass correlation coefficient

### Table S10. Correlation between participant 6-month characteristics and total completion of *Dos*

| **Predictors** |  | **Correlation coefficient, r (*P* value)** |
| --- | --- | --- |
| Characteristics of participants |  |  |
| Age^a^ |  | -0.07 (.60) |
| Gender^b^ |  | 0.06 (.64) |
| Highest level of education |  | 0.12 (.35) |
| ActiGraph^a^ |  |  |
| SB *(mins per day)* |  | -0.15 (.25) |
| Percentage of SB per day *(SB per wear time)* |  | -0.22 (.10) |
| Average duration of SB bouts *(mins)* |  | 0.08 (.56) |
| Number of SB bouts per day |  | -0.15 (.26) |
| Number of SB breaks per day |  | -0.15 (.26) |
| MVPA *(mins per day)* |  | 0.21 (.11) |
| LPA *(mins per day)* |  | 0.14 (.30) |
| VM *(counts per day)* |  | 0.23 (.08) |
| Steps *(per day)* |  | 0.14 (.31) |
| AQoL-6D Utility instrument^a^ |  | 0.18 (.17) |
| HADS^a^ |  |  |
| Anxiety |  | -0.12 (.35) |
| Depression |  | -0.15 (.26) |

^a^Pearson’s correlation.

^b^Spearman’s rho.

SB, sedentary behavior; MVPA, moderate-to-vigorous physical activity; LPA, light-intensity physical activity; VM, vector magnitude HADS, Hospital Anxiety and Depression Scale.

### Table S11. Smartphone app usability, the Unified Theory of Acceptance and Use of Technology (UTAUT2) questionnaire

| **Construct (7-point Likert scale)** | **6-month**  **(n=39)** |
| --- | --- |
| Performance expectancy | 4.03 (1.71) |
| Effort expectancy | 5.50 (4.00-6.50) |
| Social influence | 4.00 (3.00-5.00) |
| Facilitating conditions | 5.50 (4.75-6.00) |
| Hedonistic motivation | 4.33 (3.67-5.67) |
| Habit | 3.54 (1.84) |
| Behavioral intention | 4.00 (1.00-7.00) |
| Use | 3.39 (1.66) |

Likert scale: 1 = strongly disagree; 4 = neutral; and 7 = strongly agree.

Reported as means (SD) or median (interquartile range) as appropriate.

### Table S12. Components of the intervention costs (Aus $)

|  | **Total cost (Aus $)** | **Average cost per participant (Aus $)** |
| --- | --- | --- |
| **Intervention costs** |  |  |
| Fitbit Inspire wearable activity tracker | $6,030.00 | $100.50 |
| Vire app and ToDo-CR program^a^ | $56,867.97 | $947.80 |
| Training and familiarization of CR clinician with the Vire app by another clinician^b^ | $564.08 | $9.40 |
| **Implementation costs** |  |  |
| Face-to-face provision of Fitbit and paper instructions to download app (CR clinician time^c^) | $510.00 | $8.50 |
| Single phone contact support cost (CR clinician time^c^ + call cost^d^) | $943.95 | $10.50^e^ |
| Attempted call minutes (CR clinician time^c^) | $13.60 | $0.23 |
| Single email contact support cost (CR clinician time^c^) | $263.50 | $5.95^f^ |
| **Total** | $65,193.10 | $1,086.55 |

^a^ Cost breakdown: Building and testing of Vire app and associated platforms = $12,000; Server maintenance across 18-months = $35,000; Additional costs of server maintenance and support due to 6-month extension of trial due to COVID-19 pandemic = $9,867.97

^b^ Eight CR clinicians trained ^b^ x 60-minutes each at three CR sites + trainer clinician time 60-minutes each at three CR sites

^c^ An ACT Health Registered Nurse Classification Level 2 wage (2020-2022) of $95,685-$107,000 was used equating to approximately $51.28/hour

^d^ Call costs based on pre-paid national call rate of $0.20/minute (2020-2022)

^e^ Based on average call length of 10-minutes

^f^ Based on average time to send one email of 7-minutes

### Table S13. Mean healthcare usage costs at 6- and 12-months

| **Healthcare usage costs** | **Baseline to 6-months** | | |  | **Baseline to 12-months** | | |
| --- | --- | --- | --- | --- | --- | --- | --- |
|  | **Intervention**  **Mean (SD)** | **Control**  **Mean (SD)** | **Mean difference**  **(Int – Con, 95% CI)** |  | **Intervention**  **Mean (SD)** | **Control**  **Mean (SD)** | **Mean difference**  **(Int – Con, 95% CI)** |
| All-cause non-elective hospital admissions, mean *(Aus $)* | 361.00 (1,287.94) | 1,111.48 (5,948.69) | -750.48 (-2,306.51 to 805.55) |  | 1,826.55 (11,454.49) | 1,327.02 (6,007.08) | 499.53 (-2,807.09 to 3,806.16) |
| Cardiac-related non-elective hospital admissions, mean *(Aus $)* | 138.70 (413.12) | 767.50 (5,838.20) | -628.80 (-2,125.08 to 867.48) |  | 252.40 (784.37) | 859.38 (5,850.84) | -606.98 (-2,116.14 to 092.18) |
| Emergency department presentations, mean *(Aus $)* | 315.83 (605.23) | 136.00 (326.61) | 179.83 (3.46 to 356.20)^a^ |  | 593.98 (1,046.12) | 312.15 (686.72) | 281.83 (-38.61 to 602.28) |
| Cost of the intervention | 1,086.55 (20.64) | 0 | 1,086.55 (1,081.22 to 1,091.88) |  | 1,086.55 (20.64) | 0 | 1,086.55 (1,081.22 to 1,091.88) |

^a^Independent samples t-test two-sided *P* = 0.04

CI, confidence intervals

Footnote: The Independent Health and Aged Care Pricing Authority Australian version of the National Weighted Activity Unit (NWAU) calculators were used to obtain hospital cost information. For the private hospital, the NWAU v16 calculator was used based on the AR-DRG v8 classification framework. For the public hospitals, the NWAU v22 calculator was used based on the AR-DRG v10 classification framework. To calculate the cost of a single non-elective admission the following information was input: hospital characteristics (i.e., intensive care unit or specialized children’s hospital, Australian State); patient and episode characteristics (Indigenous status, funding source, length of stay and intensive care hours); residential remoteness; AR-DRG classification; hospital-acquired condition adjustments (i.e., age, sex, emergency status, transfer status). To calculate the cost of a single ED presentation the NWAU v20 calculator for ED services was used and the following information was input: URG classification; age; compensable status; and Department of Veterans Affairs status. Costs associated with medication and primary care physicians were not collected. A discount rate was not applied given the 12-month timeframe examined.
